# The Role of Physical Activity on Spatial and Temporal Cognitive Processing in Young Women

**DOI:** 10.3390/brainsci15050431

**Published:** 2025-04-23

**Authors:** Joaquín Castillo-Escamilla, María del Mar Salvador-Viñas, José Manuel Cimadevilla

**Affiliations:** 1 Department of Psychology, University of Almería (UAL), 04120 La Cañada, Spain; adx94@yahoo.es (J.C.-E.); mariasv1798@gmail.com (M.d.M.S.-V.); 2 Health Research Center, University of Almería (UAL), 04120 La Cañada, Spain; 3 Faculty of Health Sciences, International University of La Rioja (UNIR), 26006 Logroño, Spain

**Keywords:** time comparison, navigation, hippocampus, virtual tasks, cognition, learning

## Abstract

**Background/Objectives:** Physical activity (PA) has many benefits for both physical and cognitive health. It has also been related to improvements in memory and executive functions. However, its impact on time estimation remains less explored. Time is a key component of episodic memory, which also involves spatial components to give a full context to events. Given the clear evidence of the benefits of PA in spatial navigation and the anatomical overlap with temporal estimation through the hippocampus, the latter could be affected in a similar way. Therefore, this study aimed to check how PA can influence time processing and spatial memory. We wanted to check if PA influenced time and space with the same directionality. **Methods:** Forty-two (*n* = 42) female university students participated in this study, divided into a Sport (*n* = 25) or Sedentary (*n* = 17) Group depending on their participation in PA for a minimum of 3 h a week. They were addressed in two different cognitive capabilities. The first was time processing, measured by the Time Comparison Task, which controlled for several key aspects of time literature in its design. Moreover, we measured spatial navigation skills, using a well-proven virtual spatial navigation task, The Boxes Room. Accuracy and mean response times were registered per task. **Results:** Significant correlations were observed between spatial and temporal task performance. In addition, PA influenced spatial and time processing in a similar way, with the Sport Group outperforming the Sedentary Group in accuracy and response times for both tasks. **Conclusions:** These findings provide evidence that PA influences time processing similarly to its established effects on spatial memory, which could help developing sports programs that further enhance this skill.

## 1. Introduction

Physical activity (PA) is widely considered a key component of an optimal state of health. It is a category that can include many activities, such as sports practice, alongside leisure activities, planned exercises, and dancing, having in common the involvement of a body movement with the contraction of skeletal muscles [1]. There is a considerable international impulse to promote a more active lifestyle in the population, as it can successfully prevent many chronic diseases and the consequences of a sedentary lifestyle [2,3]. Among the many benefits of PA, we can find a reduced risk of developing many organic diseases such as hypertension, obesity, and diabetes, among others [4,5]. PA can also help reduce the mental health risks for conditions such as depression [1].

However, the benefits are not only focused on preventing disease or improving the fitness of the body. A physically active lifestyle can also help enhance brain function in several populations. There are meta-analyses reporting a general benefit of PA in cognitive processes such as attention, motor control, or visuospatial memory [6,7]. Other studies show benefits in executive control [8], motor inhibition, and cognitive flexibility [9].

Moreover, PA results in clear improvements in learning tasks that are dependent on hippocampus learning, thus demanding the encoding of spatial signals that can form a cognitive map of the environment [10]. Therefore, physically active individuals are able to outperform sedentary participants in spatial navigation tasks in both animals [11,12] and humans [13,14,15]. This also extends to the implementation of training programs on untrained individuals. A review [16] identified improvements in several tasks related to navigation and orientation after physical training. Advancements in technology have fostered progress within this field of study, specifically through the development of virtual spatial navigation tasks. A key advantage of these tasks, relative to traditional spatial measures, is their enhanced ecological validity, attributed to their capacity to assess cognitive skills pertinent to real-world navigation [17]. These tasks were able to find clear reliable differences due to PA in previous studies [13,14].

However, among cognitive functions and how PA can affect them, the processing of time is less studied. Considering the possible models of its functioning, clock timing theories [18] state that time pieces are stored in working memory, which works in unison with long-term memory to make temporal judgments. Other authors consider time to be widespread in several regions that are involved depending on the demand [19,20]. Interestingly, there is more evidence that might indicate an influence of PA on time processing, which is related to its anatomical overlap with spatial processes. Some authors relate the hippocampus to time processing [21], and the entorhinal cortex is also involved [22], which are both relevant structures to spatial memory [23]. Furthermore, the hippocampal place cells that fire in response to exploring the environment [24,25] can also act as time cells, encoding the particular chronology of events alongside their placement in space [26]. Additionally, working memory, a key component of time processing, is also said to influence allocentric spatial navigation [27]. Thus, and given the extensive literature in this matter, any potential benefit of PA on spatial cognition should follow the same pattern and directionality for time perception and processing due to these anatomical and functional overlaps.

A potential difficulty in properly revealing time processing and the mediator role of PA is related to the methodology of the tasks involved in its assessment, on the contrary to spatial memory assessment. Some of the studies mentioned above do not purely measure the time separated from other processes, which could influence the outcome [28]. Thus, developing a purer measure of strict time-related processes, with minimal cross-cognitive processing, may be beneficial in further disclosure of these tendencies. On this note, there are three general ways to measure time: by comparing intervals, producing or reproducing a previous duration, or a more subjective estimation [29]. Most experimental procedures do not differentiate durations above and below 1000 ms, which are known to be processed differently processed in the brain [30]. On this note, time intervals above the realm of a second involve more cognitive processing [31]. Moreover, particularly for comparing intervals, the minimum duration should be 200 ms, and the difference between intervals at least 100 ms, to avoid misjudgments of time [32]. Additionally, stimuli and motion can also induce additional cognitive biases [33,34]. Lastly, faster stimuli—when compared to a reference interval—are processed differently in the brain versus slower stimuli [35,36,37,38,39]. Thus, any task that tries to properly measure time should consider this evidence. In this respect, some authors used many different time intervals successfully [40], but applied to auditory stimuli. To our knowledge, all these parameters, taken together, have not been applied to a unitary computerized task design.

This information highlights several key findings. Firstly, physical activity (PA) demonstrates beneficial effects on multiple cognitive functions, notably spatial processing. Secondly, there is evidence for shared neuroanatomical substrates underlying spatial and temporal processing. Thirdly, despite this neural overlap, temporal processing and the influence of PA upon it represent a comparatively understudied area. Lastly, this research gap may correlate with a deficiency in validated experimental tasks for assessing temporal cognition, in contrast to the more established methodologies within the spatial domain. Thus, the main goal of this study is to address how physical exercise (PA) can influence time processing and if its directionality is coincidental to spatial processing. We assessed the latter component with The Boxes Room Task [41], a virtual, active, hippocampal-based navigation task. The task has demonstrated clear PA-related differences in humans [13,14] and has solid ecological validity [17]. Thus, it can guarantee a valid assessment of allocentric spatial processing. Regarding time processing and considering the difficulties found in the literature, we used the Time Comparison Task [42]. We chose this task because it offered a design covering several key aspects that could influence time processing, as stated in the prior paragraph. Based on the time comparison paradigm [29], it was designed to control the influence of the nature of the intervals by presenting stimuli with durations above and below 1000 ms [30] and faster/slower comparisons [36]. Furthermore, the intervals also differed in more than 100 ms, were at least 200 ms in duration, and lacked any motion to avoid cognitive biases [32,33,34]. To our knowledge, this is one of the few experimental procedures in time processing that controls for all these aspects simultaneously. Thus, it offers an enhanced validity compared to other alternatives in the literature.

We expected physically active individuals to outperform sedentary participants in spatial navigation measures, as reported by previous studies with the same task [13,14]. Additionally, and given the functional and anatomical overlap between spatial and temporal circuits [21,26], we expect this trend to also be present for the Time Comparison Task. That is, PA should positively influence performance and be positively related to spatial measures.

## 2. Materials and Methods

### 2.1. Participants

First, an a priori power analysis was performed G*Power v. 3.1.9.7 [43] for a repeated measures ANOVA (within–between interaction) with a = 0.05, power = 0.95, f = 0.253, 2 groups, and 4 measurements. The total required sample size was N = 36. We opted for f = 0.253 as it represents a medium effect, equivalent to eta partial squared of 0.06, following Cohen’s conventions [44].

This study was carried out by *n* = 42 female volunteer students from the University of Almera (M age = 19.85; SD = 4.25). They were divided into two groups depending on whether they were physically active (Sport Group) or not (Sedentary Group). The Sport Group consisted of *n* = 25 participants (M age = 19.60; SD = 3.24) and the Sedentary Group consisted of *n* = 17 participants (M age = 20.23; SD = 5.50). Participants were included in the Sport Group if they practiced aerobic sports for a minimum of 3 h a week. This followed the criteria of a previous study with a similar spatial assessment [13]. On the contrary, the participants assigned to the Sedentary Group did not perform any physical exercise in the same time and neither did they for several years before it. Various exclusion criteria were considered, such as excessive consumption of alcohol and/or other drugs, having suffered a head injury, or having a formal diagnosis of any type of psychological deficit or disorder. These criteria were chosen due to their influence on spatial performance, as stated in multiple references in the field [45,46,47,48]. Furthermore, participants who had any type of vision deficit were required to wear the appropriate glasses or contact lenses at the time of evaluation, as vision can impact performance in sports contexts [49].

Before starting the evaluation, each participant was informed about the main objective of the study and their freedom to withdraw at any time during the evaluation. Furthermore, this study was approved by the Ethics Committee of the University of Almera and its methodological design and procedure followed the requirements of Directive 2001/20/EC of the Council of European Communities and the Declaration of Helsinki for biomedical research involving human subjects.

### 2.2. Materials

#### 2.2.1. Time Comparison Task [42]

**Figure 1:**
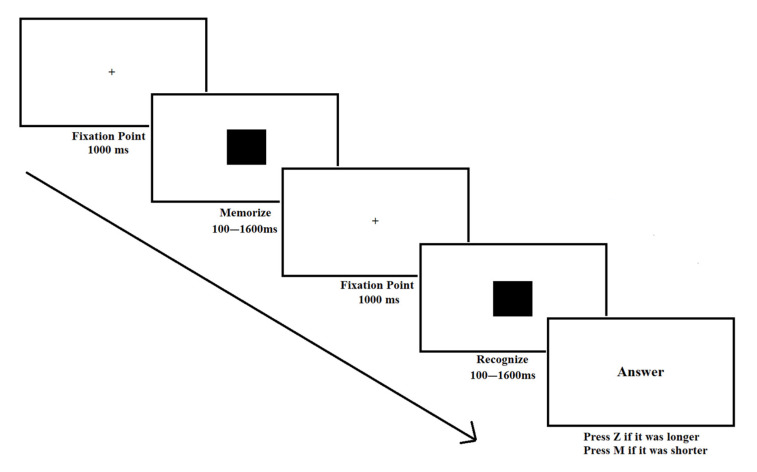
Sample trial of the Time Comparison Task The participants needed to decide if the second square’s presentation was faster or slower than that of the first.

To begin, the participants performed the Time Comparison Task. It was designed using PSNet e-Prime software (version 3.0.3.80) and participants performed it on a Hewlett Packard (HP) 3.60 Ghz computer, equipped with 16 GB RAM and a 17-inch thin film transistor (TFT) screen (1360 × 768 pixels). For this task, a fixation dot appeared for 1000 ms (see Figure 1) in the center of the screen. Afterward, a black square appeared, which remained fixed on the screen for a variable duration (200, 400, 600, 800, 1000, 1200, 1400, or 1600 ms). After this period, the fixation dot reappeared during 1000 ms, followed by a new screen showing the same black square for a variable duration. When the square disappeared from the screen, participants had to respond whether the second square had remained on the screen for a longer or shorter duration relative to the first. If they thought the duration was longer, they had to press the Z key on the keyboard; if they thought it was shorter, they had to press the M key. A total of 12 trials were presented, 6 per type and category of trials, following the recommendations of Miller et al. (2024) [50]. Trials in this task were designed to control for several relevant aspects from the time processing literature, as stated in the introduction. The accuracy and response times were individually recorded for each possible time combination. These combinations are reported in Table 1.

#### 2.2.2. The Boxes Room Task [41]

**Figure 2:**
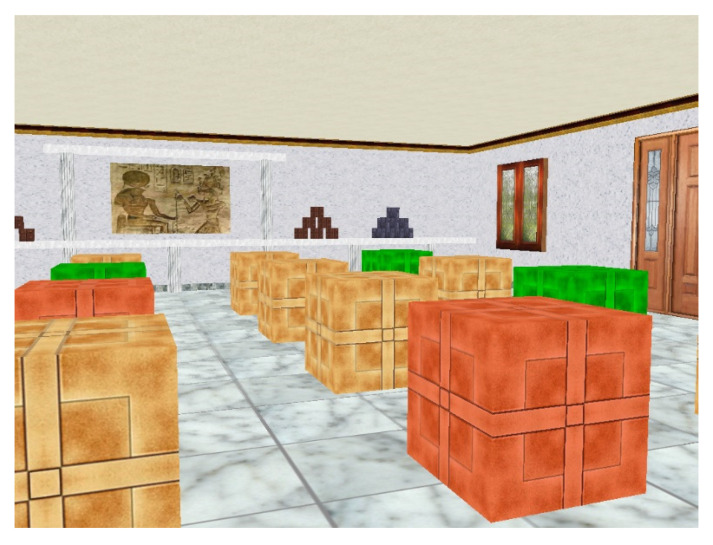
Sample trial of the Boxes Room. The participants needed to find the correct boxes (outlined in green) and avoid the incorrect ones (outlined in red) by moving inside the room.

The participants then performed The Boxes Room Task [41]. It was performed on a 2600 MHz Hewlett Packard laptop, sourced from Palo Alto, CA, United States, equipped with 3 GB of RAM and a 15.4″ TFT XGA color screen (1920 × 1200 pixels). It also provided auditory and visual feedback. This was a non-immersive virtual reality (VR) test where participants were presented with a three-dimensional room with 16 brown boxes symmetrically distributed on the floor. In addition, on the walls of the room were present various visual references, such as a door, a window, and different images of artistic works, which disambiguated the spatial locations (see Figure 2). To move around the room, they had to use a Logitech Attack 3 joystick connected to the laptop using a wired USB connection.

The main objective of this task was to find the five award-winning boxes in each of the 10 trials presented. To do this, using the joystick, they had to approach the boxes until they turned blue, indicating that by pressing the trigger they could open it. If a prize-winning box was opened, the color changed to green and they could hear a pleasant melody. If, on the other hand, a wrong box was opened, it turned red and no melody was heard. During the trial, the opened boxes remained the corresponding color until the participants had found all the rewarding boxes or until 150 s had elapsed. The reward boxes remained in the same locations during all trials, allowing the subjects to perform better from one trial to the next. Additionally, the starting point was changed between trials to avoid egocentric solutions. Participants were asked to try to find the green boxes as quickly as possible and to avoid opening the red boxes. They were not informed about the spatial strategies, the change of the starting position, or the position of the rewarding boxes. Accuracy and latency per trial were registered.

### 2.3. Procedure

Subjects were individually tested in the laboratories of the University of Almera. Before beginning the computerized tests, they were informed about the objective of the study and signed an informed consent. The researcher then conducted a brief computerized interview to record possible exclusion criteria. They were then placed in individual soundproof experimental rooms to avoid possible distractions. The evaluator explained the instructions for the Time Comparison Task [42], leaving the room once the participant had understood the procedure and completed the practical tests. After this test, the same procedure was followed with The Boxes Room Task. The entire experiment lasted between 20 and 25 min.

### 2.4. Data Analysis

In the Time Comparison Task, the percentage of correct responses (accuracy) and the response time (RT) were chosen as dependent variables, following prior works that used ePrime for related cognitive capabilities such as working memory [51]. Following the time literature stated in the introduction, these variables were further divided by the type of trial (type; faster or slower) and the time difference (time; above and below 1000 ms). On the other hand, in The Boxes Room Task, the mean error and latencies were chosen as dependent variables. In this case, the means per trial were averaged by block, from trial 2 to 4, from 5 to 7, and finally from 8 to 10, following previous studies with this task to avoid error dispersion [27]. Trial 1 was discarded for the averaging for errors and latencies, as participants still did not identify the positions of the prized boxes, having a chance-level performance. After classifying these variables, the analyses were performed.

- First, for the Time Comparison Task, two repeated measures ANOVAs (group (athlete/sedentary) × type (faster/slower) × time (above/below 1000 ms)) were performed; the first was based on precision scores, while the second used response times as dependent variables.
- Then, for The Boxes Room Task, two separate repeated measures ANOVAs (group (athlete/sedentary) × block (2–4/5–7/8–9)) were performed. In this case, the mean errors per block were used in the first ANOVA, while the mean latency per block was used in the second.
- Lastly, Pearson correlations were conducted for all combinations of the variables detailed in the prior paragraph to check internal validity in both tasks alongside the relationship between spatial and temporal measures. Additionally, relevant correlations between tasks were further checked using separate linear regression analyses.

In all cases, post hoc tests were applied using the Bonferroni procedure for interactions with a significance level of *p* < 0.05. Analyses were performed with IBM SPSS Statistics (version 25).

## 3. Results

### 3.1. Time Comparison Task—Accuracy and Response Times per Group

For the mean precision in the Time Comparison Task, repeated measures ANOVA showed a main effect on time (F(1,40) = 23.48; *p* = 0.000; η_p_^2^ = 0.370) and type (F(1,40) = 57.77; *p* = 0.000; η_p_^2^ = 0.591), but not on group (F(1,40) = 1.69; *p* = 0.201). The interactions group × type (F(1,40) = 5.66; *p* = 0.022; η_p_^2^ = 0.124) and time × type (F(1,40) = 35.01; *p* = 0.000; η_p_^2^ = 0.467) reached significance.

**Figure 3:**
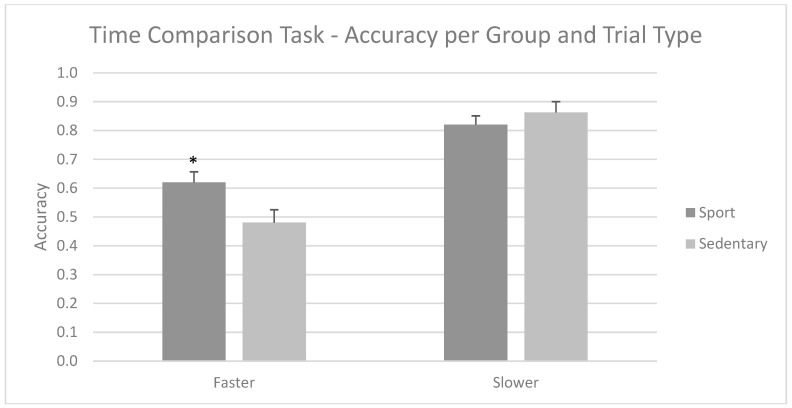
Mean accuracy scores for the Time Comparison Task per type and group of tests. The Sport Group was better than the Sedentary Group for faster trials. Mean + SEM. * *p* < 0.05.

When assessing the interaction of group × type with post hoc analyses (Bonferroni procedure), there were different patterns regarding the type of the trial. For faster trials, the Sport Group had better precision (M = 0.62; SD = 0.19) than the Sedentary Group (M = 0.48; SD = 0.16), with *p* = 0.021. For slower trials, both groups were similar (*p* = 0.384). Both groups performed better in slower trials compared to faster trials. This interaction result is shown in Figure 3.

**Figure 4:**
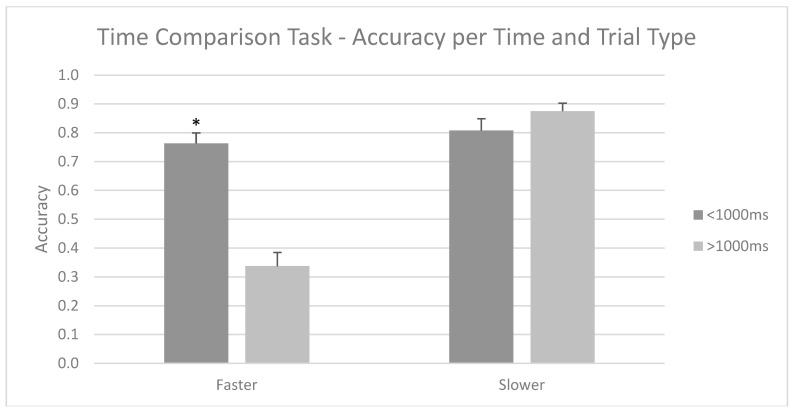
Mean accuracy scores for the Time Comparison Task per type and time of trial. For faster trials, the above 1000 ms trials were significantly worse in accuracy, for both below 1000 ms faster trials and above 1000 ms slower trials. Mean + SEM. * *p* < 0.05.

Furthermore, regardless of group, post hoc analyses of interaction Type × time showed that, for trials greater than 1000 ms, faster trials were significantly worse (M = 0.33; SD = 0.30) than slower trials (M = 0.87; SD = 0.17). Furthermore, for faster trials, those below 1000 ms showed better accuracy (M = 0.76; SD = 0.24) compared to those above 1000 ms (M = 0.33; SD = 0.30). This is shown in Figure 4.

**Figure 5:**
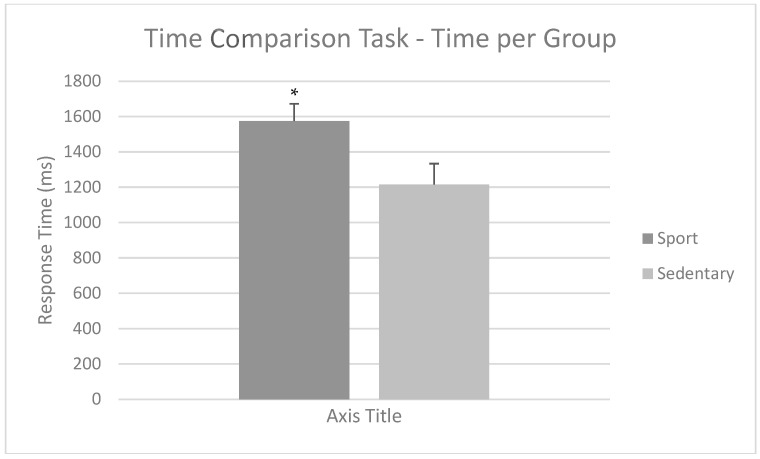
Mean response times for the Time Comparison Task per group. The Sport Group was slower than the Sedentary Group. Mean + SEM. * *p* < 0.05.

When comparing response times on this task, repeated measures ANOVA showed a main effect of type (F(1,40) = 5.66; *p* = 0.022; η_p_^2^ = 0.124) and group (F(1,40) = 5.42; *p* = 0.025; η_p_^2^ = 0.120), but not on time (F(1,40) = 3.22; *p* = 0.080). There were no statistically significant interactions (*p* > 0.050). Regarding the group factor, the Sport Group was slower (M = 1573.94; SD = 558.74) than the Sedentary Group (M = 1215.08; SD = 362.97). This is shown in Figure 5. Regardless of the group, faster trials needed more response time (M = 1573.94; SD = 685.36) than slower trials (M = 1215.08; SD = 586.28).

### 3.2. The Boxes Room Task—Errors and Response Times per Group

**Figure 6:**
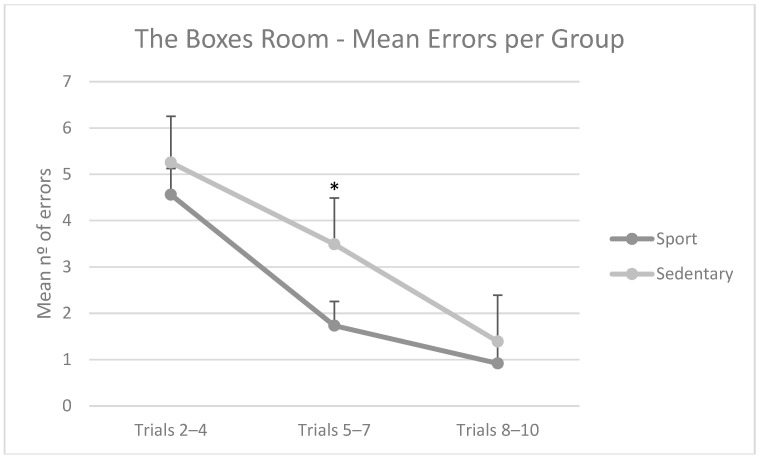
Mean number of errors per block of trials per group. The Sedentary Group had more errors in the second block (trials 5–7) compared to the Sport Group. Moreover, the Sport Group learned the task in fewer trials. Mean + SEM. * *p* < 0.05.

Regarding mean errors in this task, repeated measures ANOVA showed a major effect in block (F(2,39) = 34.28; *p* = 0.000; η_p_^2^ = 0.637), but not in group (F(1,40) = 2.12; *p* = 0.152). However, the block × group interaction was statistically significant (F(2,39) = 3.27; *p* = 0.048; η_p_^2^ = 0.44). Post hoc analyses of this comparison of differences between groups showed that participants in the Sedentary Group had more errors in the second block of trials (M = 3.49; SD = 2.94) compared to the Sport Group (M = 1.73; SD = 2.37). Both groups performed similarly in the other trials. Additionally, there was a different intragroup pattern. The Sedentary Group improved its performance through the task, in which each block had fewer errors than the former, while the Sport Group improved from block 1 to 2, but not from 2 to 3. These results are depicted in Figure 6.

**Figure 7:**
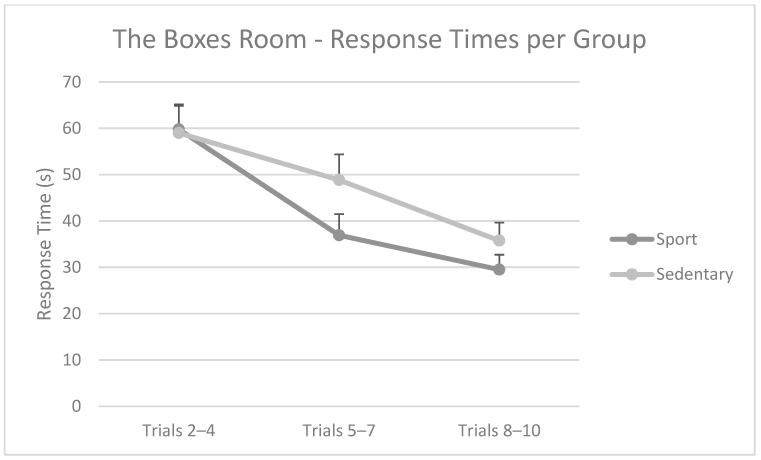
Mean response times per block of trials per group. There were no differences between groups. In intragroup differences, the Sedentary Group became faster through each trial block gradually. In contrast, Sport Group was able to get faster only in trials 2–4 and 5–7. Mean + SEM.

When considering mean latencies separately, repeated measures ANOVA showed a similar pattern, with a main effect in the trial (F(2,39) = 30.02; *p* = 0.000; η_p_^2^ = 0.606), but not in the group (F(1,40) = 1.00; *p* = 0.322. The interaction block × group was significant (F(2,39) = 3.84; *p* = 0.030; η_p_^2^ = 0.65). Post hoc analyses showed that, as shown in Figure 7, while there were no differences between the groups between the Sport and Sedentary groups, there was a different intragroup pattern. The response times of the Sport Group only improved from block 1 (trials 2–4) to block 2 (trials 5–7), while the Sedentary Group improved gradually through all blocks.

### 3.3. Correlations and Linear Regressions (Full Sample; n = 42)

Regarding significant correlations between tasks, it was shown that accuracy in faster trials of the Time Comparison Task was negatively correlated with the latency of The Boxes Room Task for two blocks, trials 5–7 (r = −0.370; *p* = 0.016) and trials 8–10 (r = −0.316; *p* = 0.042). Additionally, the precision in trials greater than 1000 ms on the Time Comparison Task was negatively correlated with mean errors in trials 5–7 of The Boxes Room Task (r = −0.320; *p* = 0.039). Both tasks showed a general pattern of internal consistency separately, with most variables of the same task relating between them at *p* < 0.050. Table A1 shows the complete correlation chart.

The significant correlations between tasks were further checked with linear regression analyses in order, showing the following patterns:

- Faster trials of the Time Comparison Task compared with the latency of trials 5–7 of The Boxes Room Task: The results showed a statistically significant model (F(1,41) = 6.36; *p* = 0.016) with an adjusted R^2^ of 0.116. This means that faster trial accuracy accounted for 11.6% of the variance of the latency in the second block of The Boxes Room Task.
- Faster trials of the Time Comparison Task compared with the latency of trials 8–10 of The Boxes Room Task: The results showed a statistically significant model (F(1,41) = 4.43; *p* = 0.042) with an adjusted R^2^ of 0.077. This means that faster trial accuracy accounted for 7.7% of the variance of the latency in the third block of The Boxes Room Task.
- Above 1000 ms trials of the Time Comparison Task compared with the mean errors of trials 5–7 of The Boxes Room Task: The results showed a statistically significant model (F(1,41) = 4.57; *p* = 0.039) with an adjusted R^2^ of 0.080. This means above 1000 ms trials accuracy accounted for 8.0% of the variance of the mean errors in the second block of The Boxes Room Task.

## 4. Discussion

The present study evaluated the influence of physical activity (PA) on spatial and temporal cognition. To do so, we used The Boxes Room Task [41] for the spatial component and the Time Comparison Task for the time processing measurement. We expected a similar influence of PA on both tasks, which provides overlapping of time–space networks [21,26]. To do so, we compared *n* = 42 female university students, divided into two subsamples (sport or sedentary group) according to their participation in PP, according to criteria from a previous study criteria using the same task [13]. The objectives were checked by Pearson correlation and several repeated measures ANOVA per task.

Our correlation and regression analysis confirmed the connection between spatial and temporal measures, as accuracy in time processing was associated with faster performance in spatial demands, alongside a smaller number of errors in some blocks of trials. Regressions were consistent with this pattern, with performance on one task being able to predict some of the score variance on the other. These results confirm the general trend in the literature in the field presented in the Introduction. That is, how spatial and temporal processes are related in the brain, resulting in overlapped performance.

It should be noted that the hippocampus is a key structure to understand the connection between these two processes. Its role in episodic memory, focused on recollection of personal experiences, is well known [52]. According to Tulving [53], events in episodic memory are encoded in a particular spatial–temporal context: What, where, and when they happened. This allows us to arrange and associate them in a coherent manner for their recovery, which depends on the medial temporal region where the hippocampus is located [54]. This proposed function is consistent with the presence of cells in this structure that can selectively fire on spatial and temporal cues [26] and its involvement on time components such as duration estimation or temporal reordering [55]. Furthermore, patients with hippocampal damage exhibit impairments when making both several time- [56] and space-related judgments [57,58]. Thus, it is not surprising to find performance similarities between our tasks.

However, not all correlations between both tasks were significant. Despite their clear entanglement, space and time processing can be partially different and thus not perfectly overlapped in performance and the influence of PA on each of them [59]. This is consistent with our result pattern. However, internal consistency in both tasks was solid, with some non-significant differences related to the differential nature of time processing in the brain depending on speed or interval duration [30,36].

When focusing on The Boxes Room Task, we found that physically active participants outperformed sedentary participants in their mean number of errors. A differential intragroup pattern was also found, as sedentary participants needed more time to improve their performance compared to sportspeople. This latter group was able to achieve a minimal number of errors after a reduced number of trials and was able to reduce their response times in fewer trials than sedentary individuals. However, both groups were able to eventually learn the task, differing in their pace and accuracy in doing so.

This general pattern of results is consistent with previous studies with these types of virtual navigation tasks, where athletes outperformed sedentary individuals in navigation-involved performance [13,14,15]. On this note, it is well known that the hippocampus shows an increased volume after the practice of physical exercise, associated with an increase in BDNF levels [60,61] and gray matter volume [62] in this region. Given the hippocampal relationship with the allocentric spatial strategy, this means that the participants were able to better encode the relationship between the different cues in the room [63,64,65]. Ultimately, this can facilitate the correct localization of target stimuli regardless of orientation, enhancing the formation of a cognitive map [25]. This can help to explain why the PA group was able to reduce its number of errors more quickly than the sedentary group and why intragroup patterns differed, with the former group achieving optimal performance and speed in fewer trials.

The Time Comparison Task presented similar findings, with the Sport Group outperforming the Sedentary Group in accuracy scores. This effect was particularly focused on faster trials, which were the ones where the comparison stimuli had less time on screen than the reference stimuli. It should be noted that the literature on time processing has generally considered faster intervals to be more cognitively taxing since early works [35,36]. Specifically, there are alterations in duration estimation versus slower intervals [39]. This was described as a faster bias in some prior literature [66,67], where judgments are altered along with speed increases, which is analogous to the presentation of faster stimuli. The advantage of the PA group in faster trials could be related to improvements in several regions of the brain and cognitive functions associated with time processing, such as time cells in the hippocampus [26].

It should be noted that sedentary individuals were faster than athletes in this task, not resulting in better performance. This may be associated with a more impulsive response pattern. Regarding this, it is known to help modulate executive functions and cognitive flexibility, as stated before [8,9]. Therefore, physically active individuals would try to prioritize precision over speed, carefully comparing the intervals before making a judgment. On this note, impulsivity is associated with enhanced psychological risks in many behaviors [68]. Thus, this slower response pattern in PA individuals might be an indicator of better psychological health, which is a common benefit of physical activity, as stated in the introduction [1,4,5].

Following the clock timing theories [18], working memory would be related to the initial encoding of the interval, and long-term memory, focused on hippocampal formation, would help with the comparison. There are some studies that relate the participation of physical exercise to a better functioning of working memory [69,70]. It was also previously explained that hippocampal function is improved in sportspeople of different age groups and sport typologies [13,14,71], consistent with its involvement with time processing [21] Thus, PA could optimize the functioning of the internal clock, reducing the cognitive burden of faster stimuli and ultimately improving performance, as shown in our sample, compensating for the faster bias.

However, and contrary to our expectations, the intervals above and below 1000 ms were similar between the groups, in contrast with prior findings [31]. However, it should be noted that our sample consisted only of young women, and men have different time processing ranges [72] and anatomical differences in key brain structures such as the hippocampus [73] that might alter performance. Women generally perceive time more slowly than men, even after controlling for their sports involvement [74]. This could also affect the performance of the perceived interval duration. Thus, as this is a limitation of our study, our results should be further expanded in future works including an equivalent male sample to outline how the sex factor might influence this lack of differences.

Another additional limitation is related to the lack of sport typology and experience specification in our sample. Several studies found that there was a difference in cognitive processing between open-skill and closed-skill sport activities [71,75]. That is, PA is done in either unpredictable or predictable environments, respectively, which could differentially influence cognitive processing. Considering that time intervals in our task were not predictable beforehand, open-skill sportspeople could present an enhanced performance compared to closed-skill individuals. Moreover, exercise intensity might also be influential, as highly trained individuals might have a more accurate perception of time during exercise due to their task knowledge after many repetitions [76,77]. However, due to sample availability, this could not be addressed. Despite this limitation, the benefit of any kind of sport typology was still better than sedentarism according to our results. However, future work should further disclose participants regarding these aspects, as there are still limited studies addressing the potential influence of these variables. Lastly, in addition to having a sample only for women, it should also be pointed out that all our participants were young adults. Having a homogeneous sample can control the variations in time perception during development [78]. Moreover, due to the limited sample availability and the scope of this paper, the modulation of some aspects such as gaming [79] or academic results [80] on performance could not be directly addressed and can be explored in future works.

Despite the acknowledged limitations, this research offers insights into several important areas. Initially, it corroborates the proposed linkage between spatial and temporal processing mechanisms, possibly involving hippocampal mediation [26,56,57]. Subsequently, it replicates the documented effect of physical activity on spatial memory within a younger cohort, supporting the suitability of The Boxes Room for evaluating spatial abilities. Importantly, analogous trends were identified in temporal performance as measured by the Time Comparison Task. Engagement in physical activity appears associated with a reduced susceptibility to certain temporal judgment biases [66,67] and may foster less impulsive responses through more accurate and cautious time estimations [68]. This suggests that PA’s benefits encompass not only cognitive function but also psychological well-being, potentially contributing to an improved quality of life for physically active individuals. Given that enhanced temporal estimation precision might be linked to decreased vulnerability to specific mental health issues [81], these results further emphasize the value of promoting sustained physical activity within the population.

## 5. Conclusions

In conclusion, despite these possible limitations, our work was able to successfully disclose the benefits of PA on time processing in young female adults. These findings were comparable to those found in the spatial memory literature, which were also replicated in our study. Future work should also disclose sport typologies to see how they might influence the relationship, in addition to considering additional aspects such as the influence of age and sex factors, which can be relevant to the processing of time [74,82]. The potential influence of factors previously demonstrated to impact spatial memory, such as gaming proficiency and academic performance [79,80], warrants investigation in the context of temporal processing to elucidate the relationship between these cognitive domains. Furthermore, the integration of behavioral data with neurophysiological measures, specifically electroencephalography (EEG), holds promise for revealing underlying neural mechanisms associated with observed tendencies. Analogous to findings that have successfully linked sports engagement, EEG activity, and spatial memory [83], this combined methodological approach may provide insights into the neural correlates of time processing. Overly, this work can help future researchers better understand the similarities between space and time and how AP can play a critical role in both, ultimately resulting in improved cognitive functions, improved neuroprotection, and a better quality of life.

## Figures and Tables

**Table 1:** Trial combinations of the Time Comparison Task. They were classified in two different time categories (above and below 1000 ms) and two trial types (faster/slower).

Square Presentation Times (1st and 2nd in ms)	Time Category (Below/Above 1000 ms)	Trial Type (Faster/Slower). Second Presentation vs. First
200–400 ms	Below	Slower
400–200 ms	Below	Faster
400–600 ms	Below	Slower
600–400 ms	Below	Faster
600–800 ms	Below	Slower
800–600 ms	Below	Faster
1000–1200 ms	Above	Slower
1200–1000 ms	Above	Faster
1200–1400 ms	Above	Slower
1400–1200 ms	Above	Faster
1400–1600 ms	Above	Slower
1600–1400 ms	Above	Faster

## Data Availability

The raw data supporting the conclusions of this article will be made available by the authors on request.

## Appendix A

**Table A1:** Complete correlation graph between the Time Comparison Task (TCT) and The Boxes Room Task (BOX) for all measures.

	TCT-Global	TCT-Faster	TCT-Slower	TCT- < 1 s	TCT- > 1 s	RT-TCT-Global	RT-TCT-Faster	RT-TCT-Slower	RT-TCT- < 1 s	RT-TCT > 1 s	BOX-B1	BOX-B2	BOX-B3	RT-BOX-B1	RT-BOX-B2	RT-BOX-B3
TCT-Global	1	0.766 **	**0.582 ****	**0.682 ****	**0.736 ****	0.035	0.066	−0.103	−0.134	0.174	−0.119	−0.259	−0.160	−0.100	−0.204	−0.215
TCT-Faster		1	−0.076	**0.357 ***	**0.717 ****	−0.018	−0.067	−0.078	−0.110	0.069	−0.055	−0.294	−0.242	−0.144	**−0.370 ***	**−0.316 ***
TCT-Slower			1	**0.607 ****	0.235	0.076	0.187	−0.062	−0.068	0.183	−0.114	−0.030	0.059	0.027	0.152	0.065
TCT- < 1 s				1	0.007	0.021	0.118	−0.045	−0.055	0.082	0.028	−0.037	−0.074	−0.121	−0.063	−0.165
TCT- > 1 s					1	0.028	−0.019	−0.100	−0.132	0.163	−0.188	**−0.320 ***	−0.150	−0.024	−0.221	−0.142
RT-TCT-Global						1	**0.940 ****	**0.919 ****	**0.841 ****	**0.878 ****	−0.013	0.028	0.221	0.014	0.069	0.066
RT-TCT-Faster							1	**0.804 ****	**0.772 ****	**0.842 ****	0.022	0.010	0.224	−0.045	0.038	0.035
RT-TCT-Slower								1	**0.874 ****	**0.717 ****	0.042	0.089	0.237	0.086	0.128	0.093
RT-TCT- < 1 s									1	**0.479 ****	0.193	0.148	**0.311 ***	0.127	0.186	0.177
RT-TCT > 1 s										1	−0.191	−0.085	0.084	−0.090	−0.053	−0.050
BOX-B1											1	**0.655 ****	**0.365 ***	**0.617 ****	**0.643 ****	**0.457 ****
BOX-B2												1	**0.706 ****	**0.402 ****	**0.677 ****	**0.632 ****
BOX-B3													1	0.115	0.301	**0.764 ****
RT-BOX-B1														1	**0.782 ****	**0.364 ***
RT-BOX-B2															1	**0.535 ****
RT-BOX-B3																1

TCT = Time Comparison Task; BOX = The Boxes Room Task; s = Seconds; * *p* < 0.050; ** *p* < 0.010. Significant correlations are highlighted in bold.
